# Adoption of routine virologic testing and predictors of virologic failure among HIV-infected children on antiretroviral treatment in western Kenya

**DOI:** 10.1371/journal.pone.0200242

**Published:** 2018-11-09

**Authors:** Julie Kadima, Elizabeth Patterson, Margaret Mburu, Cinthia Blat, Margaret Nyanduko, Elizabeth Anne Bukusi, Craig Cohen, Patrick Oyaro, Lisa Abuogi

**Affiliations:** 1 Family AIDS Care and Education Services (FACES), Kenya Medical Research Institute (KEMRI), Kisumu, Kenya; 2 University of Colorado School of Medicine, Aurora, Colorado, United States of America; 3 Department of Obstetrics, Gynecology & Reproductive Sciences, University of California San Francisco, San Francisco, California, United States of America; 4 University of Nairobi School of Medicine, Nairobi, Kenya; 5 Department of Pediatrics, University of Colorado Denver, Aurora, Colorado, United States of America; Medecins Sans Frontieres (Switzerland), SWAZILAND

## Abstract

**Background:**

Access to routine virologic monitoring, critical to ensuring treatment success, remains limited in low- and middle-income countries. We report on implementation of routine viral load (VL) monitoring and risk factors for virologic failure among HIV-infected children on antiretroviral treatment (ART) in Western Kenya.

**Methods:**

Routine VL testing was introduced in western Kenya in November 2013. We performed a case-control study among 1190 HIV-infected children ≤15 years on ART who underwent routine VL testing June 2014–May 2015. A random sample of 98 cases (virologic failure define as VL >1000 cps/mL) and 201 controls (VL <1000 cps/mL) from five facilities in three high HIV prevalence counties in Kenya were followed for a minimum of 12 months. Data from patient charts were analyzed using logistic regression to determine factors associated with failure to attain virologic suppression at initial routine and subsequent VL testing among cases.

**Results:**

Overall, 1190 (94%) children with a median age of 8 years underwent routine VL testing of whom (37%) had virological failure. Among the 299 cases and controls, WHO stage, baseline CD4 count and time since ART initiation were not associated with virologic failure during the follow-up period. In multivariable analysis, unsuppressed children at initial test were more likely to be male (adjusted Odds Ratio (aOR) 2.1, 95% Confidence Interval (CI) 2.1–3.6) and have had an ART regimen change (aOR 2.0, CI 1.0–3.7) than controls. Of the two-thirds of children 201/299 who had a subsequent VL performed, VL suppression was greater among those suppressed at initial test 126/135 (93.3%) compared to children with virologic failure 15/66 (22.7%, p<0.0001). Among those failing at first test who achieved viral suppression in follow up, 12/15 (80%) were on a protease inhibitor (PI)-based regimen. In the multivariable analysis of children with subsequent VL testing, children on PI-based 2^nd^ line regimens were 10-fold more likely to achieve viral suppression than children on first-line NNRTI-based ART (adjusted Odds Ratio [aOR] 0.1; 95%CI 0.0–0.4).

**Conclusion:**

Coverage of initial routine viral load testing among children on ART in western Kenya is high. However, subsequent testing and virologic suppression are low in children with virologic failure on initial routine viral load test. There is an urgent need to improve management and viral load monitoring of children living with HIV experiencing treatment failure to ensure improved long-term outcomes.

## Introduction

In October 2014, the Joint United Nations Programme on HIV/AIDS (UNAIDS) [1] proposed ambitious new targets to end the AIDS epidemic and expand HIV treatment. The “90-90-90” goals target propose that by 2020, 90% of all people living with HIV will know their status, 90% of people diagnosed with HIV will be receiving antiretroviral therapy (ART) and 90% of people on ART will achieve viral suppression. Reaching the 90-90-90 goal for children living with HIV is particularly challenging. In 2015, an estimated 1.8 million children under the age of 15 were living with HIV but less than half were diagnosed and in care and an estimated 150,000 children are newly infected annually adding to the burden [2,3]. Amongst children living with HIV only 49% had accessed ART in 2015 [4]. Finally, lack of access to viral load monitoring and reports of low virologic suppression threaten achievement of the final “90” target for children [5].

Virologic suppression is the hallmark of successful HIV treatment in both adults and children. However, due to early immunologic damage and high viral loads found in many children, attaining rapid virologic suppression in children is critical. Children with HIV are faced with the need for lifelong ART but have limited drug options appropriately formulated for children and few alternative regimens[6–8]. Long-term viral load suppression, particularly when obtained during infancy, is associated with improved neurocognitive and growth outcomes [9,10] as well as lower viral reservoirs [11]. Several studies indicate that viral failure may be higher in children than in adults [5,12]. A recent systematic review found that virologic suppression rates for children on ART ranged between 60–75%, and this rate had only improved slightly since 2000 [5]. This is well below the rate of suppression found for adults in low- and middle-income countries (LMICs) (85%) in a similar systematic review, and far below the rate for children in high income countries (90%). Prolonged treatment with a failed regimen can lead to increased drug resistance [13,14], higher viral load and increased risk of failing second line therapy [15]. Conversely, premature switching without confirmation of virologic failure limits treatment options and leads to unnecessary increases in cost. Clinical and immunologic criteria for predicting treatment failure have low sensitivity and low positive predictive value for identifying those with virologic failure, particularly in children [16–18]. Viral load (VL) testing has thus become essential for accurate management and decision-making for children on ART.

In 2013, the World Health Organization (WHO) recommended the use of viral load to monitor treatment efficacy [19]. While most LMICs have national guidelines supporting the WHO recommendation for routine viral load monitoring, very few have implemented it on a large scale. A 2015 CDC survey among seven sub-Saharan countries found that only two countries tested >85% of patients on ART, while four countries tested <25% of patients on ART [20]. Literature on the challenges and outcomes from implementing routine viral load monitoring specifically for children in resource limited settings is scant.

In Kenya, there were an estimated 98,000 children under the age of 15 living with HIV in 2015 [21]. An impressive 77% of these children had access to antiretroviral therapy. Routine viral load monitoring was implemented in western Kenya in late 2013. Thus, we aimed to describe the adoption of routine viral load monitoring among HIV-infected children <15 years old on ART in western Kenya and evaluate factors associated with viral suppression in this previously unmonitored cohort. Findings from this study may inform the Kenyan national ART program and other similar settings on the management of children on ART.

## Methods

### Design

This was a nested case-control study conducted within routine HIV program implementation. From a cohort of 1272 children receiving ART at five Ministry of Health facilities in southwestern Kenya, 98 cases (children with VL ≥ 1000 cps/mL) were frequency matched by facility to 201 controls (children with VL < 1000 cps/mL) then followed for a minimum of 12 months to ascertain rates of subsequent testing and viral load suppression. Facilities were chosen to be representative of geographic sub-locations in the region. All facilities had access to routine viral load monitoring during the study period.

### Setting

Kenya has the fourth-largest HIV epidemic in the world, with 1.5 million people living with HIV and an adult HIV prevalence of 5.9% in 2015[21]. Homabay, Migori and Kisumu counties in Kenya, have a largely rural population and are the top contributors to the new pediatric HIV infections nationally [22]. There are an estimated 98,000 children currently living with HIV, most of whom are living in western Kenya [21,22]. Ministry of Health nurses and clinical officers (equivalent of physician assistants) provide the majority of HIV services within primary health care facilities with support from HIV implementing partners. Three sub-county hospitals and two health centers were selected for this study because of their high patient volume and location in distinct geographic areas. At the time of this study, Family AIDS Care & Education Services (FACES) was supporting implementation of HIV services at these facilities in collaboration with the county governments of Migori, Homabay and Kisumu Counties. FACES is a collaboration between the University of California San Francisco (UCSF), the Kenya Medical Research Institute (KEMRI), and the Kenyan Ministry of Health (MOH) which works to build the capacity of the Kenyan government to implement quality HIV services through targeted technical support, training, and health care workforce support [23].

The 2014 Kenya National ART Guidelines were followed throughout this study [24]. HIV infection was diagnosed through HIV DNA PCR testing for children <18 months and HIV antibody testing for children ≥ 18 months. Treatment was initiated among all confirmed HIV positive children ≤ 10 years of age and (Table 1) above age 10 meeting any of the following criteria: a CD4 count of ≤ 500cells/mm^3^, WHO Stage of III or IV, or co-infection with TB or Hepatitis B. The recommended first-line ART regimen is described by age in Table 1 below.

**Table 1:** ART eligibility and preferred drug regimens for Kenyan children 2014.

Age (years)	Eligibility Criteria	Recommended 1^st^ line Regimen	Recommended 2^nd^ Line Regimen
< 3	ALL	Abacavir+Lamivudine+Lopinavir/ritonavir	Zidovudine+Lamivudine+Darunavir/ritonavir*
> 3–10 and adolescents <35 kg	ALL	Abacavir+Lamivudine+Efavirenz	Zidovudine+Lamivudine+Lopinavir/ritonavir
>10–15 and weight ≥ 35 kg	CD4 ≤ 500cells/mm^3^, WHO Stage III or IV, HIV/TB or HIV/Hepatitis B co-infection	Tenofovir+Lamivudine+Efavirenz	Zidovudine+Lamivudine+Lopinavir/ritonavir

*Darunavir available through consultation with regional or national Therapeutic Technical Working Group for children > 3years of age. For children < 3years of age on PI-based regimen, further consultation required.

**Fig 1:**
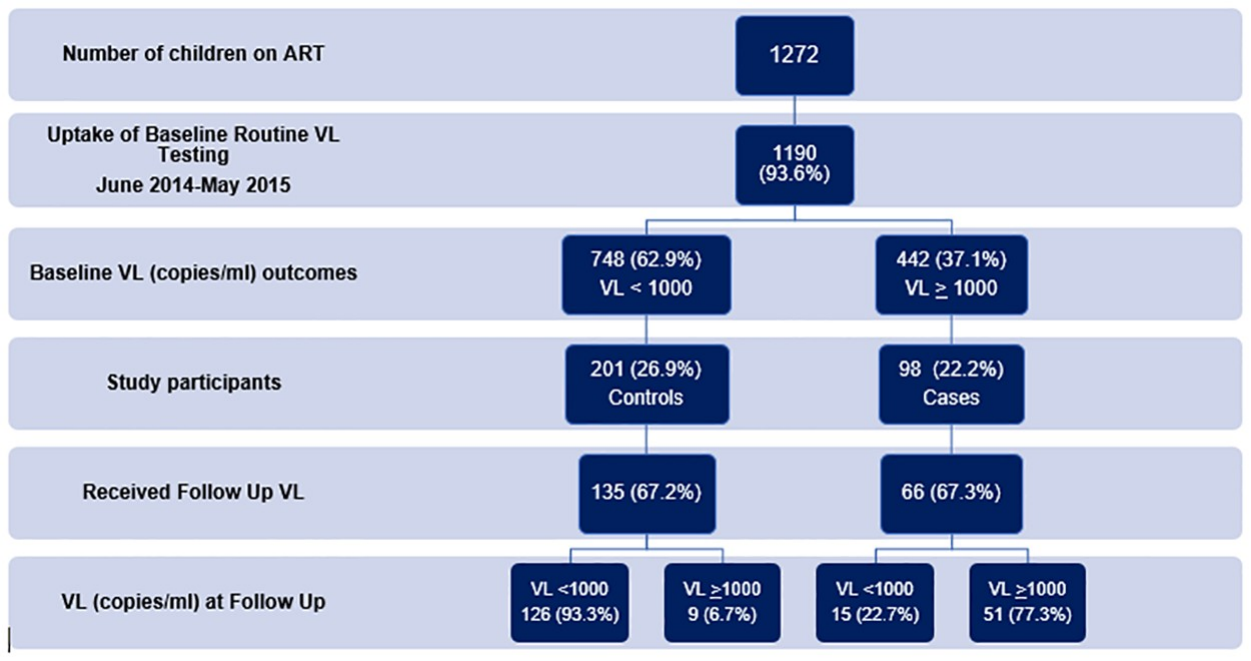
Kenya National HIV viral load monitoring algorithm 2013.

Prior to 2013, viral load was available in Kenya by request for: 1) single drug substitutions, or 2) suspected treatment failure meeting standardized criteria. In November 2013, with support from the U.S. Centers for Disease Control and Prevention, routine viral load testing was adopted in a phased approach by Ministry of Health facilities in Western Kenya. Per Kenya 2013 National HIV Guidelines, HIV positive patients (adults and children) on ART undergo viral load testing at six months post-ART initiation, at 12 months and annually thereafter. (Fig 1) Those with detectable viral load defined as ≥ 1000 copies(cps)/mL (cps/mL) are presented for a multidisciplinary team review, home visit, and intensified adherence support. (24) Subsequent viral load testing is recommended 3 months after adherence intensification interventions to confirm treatment failure. Those whose viral load remains above 1000 cps/mL are switched to second line therapy. (Table 1) All VL samples are networked to a referral testing facility through road transportation.

### Participants and sampling

Among 1,272 HIV positive children on ART ages 0–15 years at five selected sites, a total of 1,190 (93.6%) had an initial routine viral load test between June 2014 and May 2015. (Fig 1) Using a FACES viral load database, a random sample of approximately 1 case (child with VL ≥ 1000 cps/mL) at initial test was frequency matched by facility to 2 controls (child with VL < 1000 cps/mL), giving a total of 98 cases and 201 controls.

### Data collection and outcomes

Charts were reviewed and participant demographic, caregiver, and clinical characteristics manually abstracted from a standardized Ministry of Health HIV clinical encounter form completed at each clinic visit. Our primary outcomes of interest were 1) adherence to the national VL monitoring algorithm and 2) viral load suppression (defined as < 1000 cps/mL) at initial routine viral load and at end of follow up. VL samples were collected on-site and sent for central processing to a regional laboratory. Initial routine viral load result was defined as most recent viral load received between June 2014 and May 2015 and were obtained from an MS Excel extract of viral load data prepared by the CDC and validated against the patient file. At least 12 months after the initial viral load result patient charts were reviewed for subsequent viral load testing and clinical data since initial viral load test. An online electronic database of VL results maintained by NASCOP was reviewed as a secondary data source for those without a subsequent VL on file. Adherence to medications for periods related to both initial and subsequent viral load testing was documented via patient or caregiver self-report and classified by health care provider based on proportion of doses taken as good (> 95%), fair (85%-94%), or poor (<85%). Attendance at scheduled clinical visits was reviewed for the year prior to initial and subsequent viral load and any missed visits were recorded. Any visit that occurred more than 3 days after the scheduled visit in the year prior to the initial viral load test was recorded as a late visit. Disclosure of HIV status to children was documented on a separate standardized Ministry of Health form by health care providers as full, partial, or not done. Full disclosure indicates that the child was aware of his/her HIV status and that reactions and concerns from either the child or caregiver have been addressed. Partial disclosure means that the child and caregiver have begun the process of assessment and preparation in readiness for full disclosure. The child may have already been sensitized that they have an illness but not all information relevant to their HIV status and lifelong treatment has been given. Not done means that the child and caregiver have not yet begun the process of preparation for disclosure. Weight for age at viral load testing was determined and z scores categorized as follows: > -2.0: normal, -3.0 to -2.0: moderately malnourished, <-3.0: severely malnourished. Data was entered into MS Excel (Microsoft, Redmond, WA) by two authors (EP and MN) and reviewed by two additional authors (JK and LA). (S1 Dataset) Any data discrepancies were resolved by manual or electronic medical file review.

### Analytical approach

We examined sociodemographic and clinical characteristics of the 98 cases and 201 controls who underwent initial routine viral load testing at the five selected facilities. Variables potentially associated with failure to achieve viral suppression were explored, including age at time of viral load, gender, facility, orphan status, HIV disclosure, weight for age, WHO Stage, CD4 count, time since ART initiation, regimen change, regimen at the time of initial and subsequent routine viral load testing, history of TB, ART adherence, missed clinic visits, caregiver relationship and caregiver HIV status. Categorical variables were assessed using the Pearson’s chi-squared tests; medians for continuous variables were compared using the Mann-Whitney (rank sum) test, a non-parametric alternative to the independent samples T-test appropriate for skewed distributions. Univariate and multivariable logistic regression models were then fitted to identify predictors of failure to suppress at first and subsequent testing and to obtain measures of association in the form of odds ratios (OR) and 95% confidence intervals (CI). All explanatory variables with a p-value of <0.2 at bivariate analysis were considered for inclusion in the adjusted model since they were likely to be associated with the outcome. Additional variables were included *a priori*. A backward stepwise approach was used in which all covariates were included in the multivariable model and were retained in the model if their removal from the model did not improve the AIC (Akaike Information Criterion) to reduce over-fitting. Explanatory variables used in analysis of subsequent viral load were age, gender, baseline WHO Stage, CD4 at enrollment, time since ART initiation, time from initial to subsequent viral load test, regimen change, and regimen at the time of initial viral load test, missed clinic visit, history of opportunistic infections, home visit by lay health care worker since initial VL, and adherence support counseling. Data analysis was performed using Stata/SE (Stata Corp, Texas, U.S.A.) Version 12.

### Ethics

The study was conducted using routine patient and laboratory records and did not involve direct involvement with human subjects. These data are routinely collected through the Ministry of Health and FACES program. An evaluation protocol is reviewed and approved annually by the KEMRI Ethical Review Committee, UCSF Committee on Human Research, and the Associate Director for Science, Division of Global HIV/AIDS, Centers for Disease Control and Prevention. All data collected was from routinely collected data forms approved by the above ethical committees. De-identified data was obtained from medical records for analysis; consent was not required from individual patients to contribute data to the study.

## Results

### Routine viral load testing

**Fig 2:**
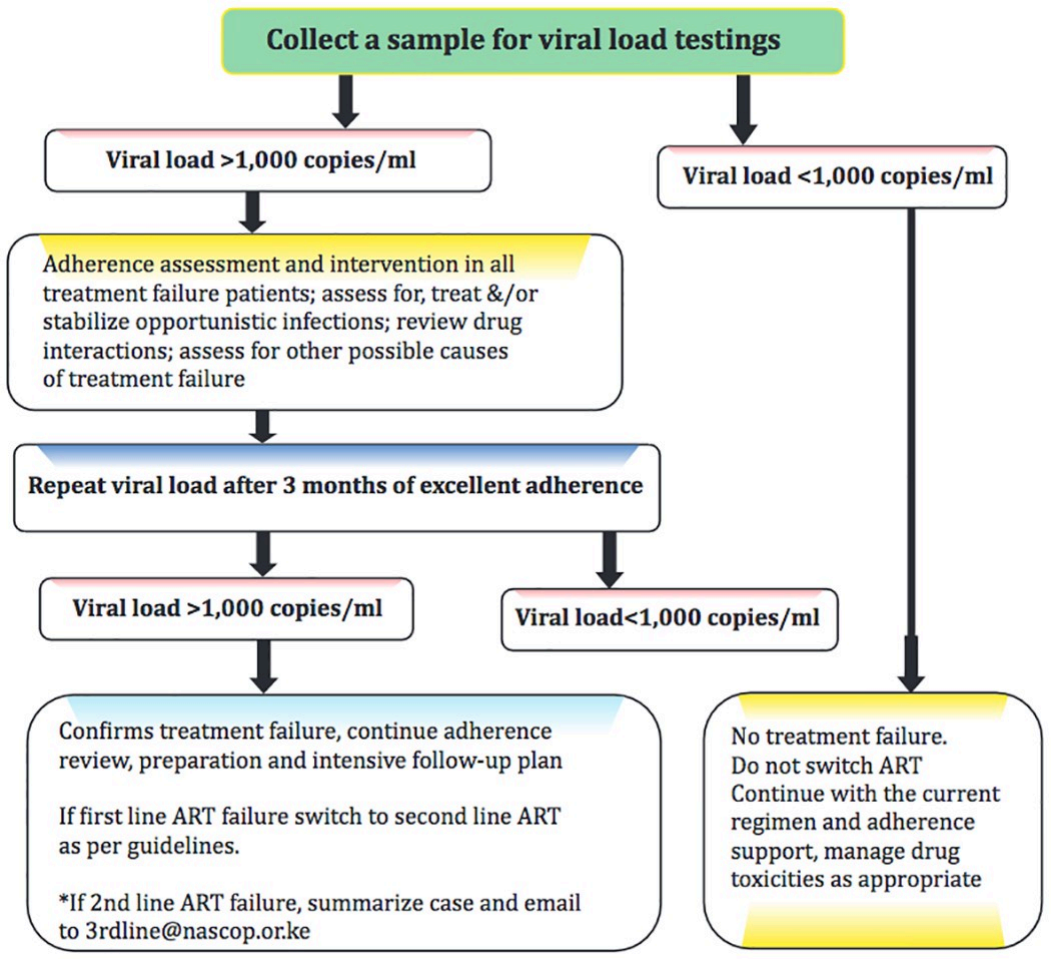
Routine viral load testing in a cohort of HIV positive children age ≤15 on ART.

Of the 1272 HIV positive children aged 0–15 years on antiretroviral treatment, 1190 (93.6%) had a initial routine viral load result, among which 442 (37%) children had a viral load ≥ 1000 cps/mL. (Fig 2) Among 98 cases and 201 controls randomly selected from this cohort, the median age was 8 years (intraquartile range (IQR) 6,10) with most children 279 (93.3%) above 2 years. Median viral load among 98 cases was 20536 cps/ml (IQR 4251, 61872) and median 0 cps/ml (IQR 0,0) in controls. Seventy-three percent (n = 208) were either WHO Stage I or II at enrollment to HIV care. Most children [n = 213 (71.5%)] were on ART for more than two years at time of initial viral load testing. The median CD4 was 680 cells/mm^3^ (IQR 420, 1170) at time of initial viral load testing. More than three-quarters of children 233 (78.2%) were on a non-nucleoside reverse transcriptase inhibitor (NNRTI)-based regimen. Most (83.3%) had parents as their caregivers and more than half (54%) had not had their HIV status disclosed to them. Ninety-five percent (n = 209) reported good ART adherence while 192 (64.4%) had missed at least one clinic visit in the twelve months prior to initial viral load testing. (Table 2)

**Table 2:** Characteristics of children on ART who were virologically-suppressed (n = 201) versus unsuppressed (n = 98) at initial routine viral load test (June 2014–May 2015).

Measure	Total (n = 299)	Unsuppressed cases (≥1000 cps/mL) (n = 98) (% or IQR)	Suppressed controls (<1000 cps/mL) (n = 201) (% or IQR)	p-value
***Sociodemographics***				
**Age (years), median (IQR)**^1^				0.23
0–2	20 (6.7)	9 (9.2)	11 (5.5)	
> 2	279 (93.3)	89 (90.8)	190 (94.5)	
**Median age**^1^ **(years) (IQR)**	8 (6,10)	8 (6,10)	8 (6,10.5)	0.89
**Facility**				0.80
Health Center 1	84 (28.1)	26 (26.5)	58 (28.9)	
Health Center 2	56 (18.7)	18 (18.4)	38 (18.9)	
Sub-county hospital 1	45 (15.1)	14 (14.3)	31 (15.4)	
Sub-county hospital 2	55 (18.4)	22 (22.5)	33 (16.4)	
Sub-county hospital 3	59 (19.7)	18 (18.4)	41 (20.4)	
**Gender**				<0.00
Female	136 (45.6)	33 (33.7)	104 (51.7)	
Male	162 (54.4)	65 (66.3)	97 (48.3)	
**Orphan Status**^1^				0.47
No	245 (89.7)	87 (91.6)	158 (88.8)	
Yes	28 (10.3)	8 (8.4)	20 (11.2)	
**HIV status disclosure to child**				0.65
No	150 (54.0)	49 (51.0)	101 (55.5)	
Full	75 (27.0)	29 (30.2)	46 (25.3)	
Partial	53 (19.1)	18 (18.8)	35 (19.2)	
***Clinical characteristics***				
**Weight for age**^2^				0.03
Normal	222 (76.8)	65 (67.7)	157 (81.4)	
Moderately Malnourished	43 (14.9)	19 (19.8)	24 (12.4)	
Severely Malnourished	24 (8.3)	12 (12.5)	12 (6.2)	
**WHO Stage**^3^				0.34
I&II	208 (73.8)	68 (73.1)	140 (74.1)	
III&IV	74 (26.2)	25 (26.9)	49 (25.9)	
**CD4 (cells/mm^3^), median (IQR)**^3^	678 (422,1173)	600 (375,1056)	700 (440,1283)	0.25
Missing	84 (28.1)	23 (23.5)	61 (30.3)	
**Time since ART initiation (years)**				0.54
< 1	26 (8.7)	6 (6.1)	20 (10.0)	
1–2	59 (19.8)	20 (20.4)	39 (19.4)	
3–9	213 (71.5)	72 (73.5)	142 (70.6)	
**Number of regimen changes**				0.01
None	212 (71.1)	60 (61.2)	152 (76.0)	
One or more	86 (28.9)	38 (38.8)	48 (24.0)	
**Current ART Regimen**^1^				<0.001
NNRTI	233 (78.2)	66 (67.4)	167 (83.5)	
PI 1^st^ Line	29 (10.1)	9 (9.2)	21 (10.5)	
PI 2nd Line	35 (11.7)	23 (23.5)	12 (6.0)	
**History of TB**				0.15
No	248 (83.2)	86 (87.8)	162 (81.0)	
Yes	50 (16.8)	12 (12.2)	38 (19.0)	
**ARV Adherence**				0.33
Good	209 (95.9)	79 (96.3)	130 (95.6)	
Poor/Fair	9 (4.1)	3 (3.7)	6 (4.4)	
Missing	79 (27.1)	16 (16.3)	63 (31.3)	
**Late to Clinic Visits**^4^				0.30
No	106 (35.6)	31 (31.6)	75 (37.5)	
Yes	192 (64.4)	67 (68.4)	125 (62.5)	
***Caregiver characteristics***				
**Caregiver Relationship**				0.67
Parent	240 (83.3)	83 (85.6)	157 (82.2)	
Relative	35 (12.2)	11 (11.3)	24 (12.6)	
Other	13 (4.5)	3 (3.1)	10 (5.2)	
**Caregiver’s HIV Status**				0.71
Positive	179 (96.2)	65 (95.6)	114(96.6)	
Negative	7 (3.8)	3 (4.4)	4(3.4)	
Missing	113 (37.8)	30 (30.6)	83(41.3)	

^1^at time of viral load testing;

^2^weight for age z scores at viral load testing z scores categorized as; ≥-2.0<z-score- Normal, -3.0<z-score to <-2.0 moderately malnourished, and z-score <-3.0 severely malnourished;

^3^at time of enrollment;

^4^in the year prior to VL test

NNRT- non-nucleoside reverse transcriptase-based regimen PI-protease inhibitor based regimen

### Risk factors for failure to suppress on initial routine VL test among children on ART

#### Univariate analysis

Children with virologic failure at initial test were more likely to be male, have moderate or severe malnutrition, and have a history of an ART regimen change. (Table 3) Male children were twice as likely to have virologic failure compared to female children (OR 2.1, 95% CI 1.3–3.5). Moderate or severe malnutrition was associated with virologic failure compared to those without malnutrition (OR 1.9, 95% CI 1.0–3.7) and (OR 2.4, 95% CI 1.0–5.7) respectively. Children with at least one medication change had twice the odds of virologic failure compared to those with no drug change (OR 2.0, 95% CI 1.2–3.4). More than one year on ART was associated with a trend toward increased odds of failure (2–3 years OR 1.7 1.7, 95% CI 0.6–4.9; >3 years OR 1.7, 95%CI 0.7–4.4). Roughly equal proportions of children on an NNRTI-based regimen (72%) and protease inhibitor (PI)-based regimen for first line (72%) were virologically suppressed while only 34% of those already on a PI-based second line treatment were suppressed. (Table 1) Odds of having viral suppression at initial test while on an NNRTI regimen were five times higher than for those on a PI regimen for second line therapy (OR 4.9, 95% CI 2.3–10.4). Children under two years of age at time of ART initiation showed a higher odds of failure but this was not statistically significant. Caregiver reported adherence was only reported as fair or poor by approximately 4% of caregivers in both cases and controls and was not associated with virologic failure. A large proportion of children had been late for at least one clinic visit in the year prior to VL test (68% cases and 63% controls) and this was also not associated with virologic failure. Caregiver relationship to child and HIV status as well as orphan status, and disclosure of HIV status to the child were not associated with virologic failure.

**Table 3:** Risk factors for virologic failure at initial viral load test among children on ART (univariate and multivariable logistic regression). N = 299.

Measure	OR (95% CI)	p-value	aOR (95% CI)	p-value
**Age (years)**				
0–2	Ref			
>2	0.6(0.2–1.4)	0.23		
**Age (years) continuous**	1.0(0.9–1.1)	0.86		
**Gender**^1^				
Female	Ref		Ref	
Male	2.1 (1.3–3.5)	0.00	2.2(1.3–3.8)	0.01
**Weight for age**^1^				
Normal	Ref		Ref	
Moderately Malnourished	1.9(1.0–3.7)	0.06	2.1(1.0–4.4)	0.05
Severely Malnourished	2.4(1.0–5.7)	0.04	3.2(1.3–7.9)	0.01
**CD4** (per 100 cell-difference)	0.98(0.94–1.02)	0.27		
**Time since initiation ART (years), n (%)**				
< 1	Ref			
1–2	1.7(0.6–4.9)	0.32		
3–9	1.7(0.7–4.4)	0.28		
**Number of regimen changes**^1^				
0	Ref		Ref	
At Least Once	2.0(1.2–3.4)	0.01	1.5(0.7–3.2)	0.27
**Current ART Regimen**^1^				
NNRTI	Ref		Ref	
PI 1stLine	1.1(0.5–2.5)	0.84	1.1(0.5–2.7)	0.82
PI 2nd Line	4.9(2.3–10.4)	<0.001	3.7(1.4–9.8)	0.01
**History of tuberculosis**^1^				
No	Ref		Ref	
Yes	0.6(0.3–1.2)	0.15	0.4(0.2–0.9)	0.02
**Caregiver Relationship**				
Parent	(Ref)			
Relative	0.87(0.41–1.87)	0.73		
Other	0.57(0.15–2.13)	0.41		
**Caregiver’s HIV Status**				
Positive	(Ref)			
Negative	1.32(0.29–6.06)	0.73		
**Late to Clinic Visits**				
No	(Ref)			
Yes	1.31(0.30–0.79)	0.30		
**ART Adherence**				
Good	(Ref)			
Poor/Fair	0.83(0.20–3.41)	0.80		
**Child disclosed to**				
No	(Ref)			
Full	1.31(0.74–2.34)	0.36		
Partial	1.07(0.55–2.08)	0.84		

ART- antiretroviral treatment, NNRTI- non-nuceloside reverse transcriptase inhibitor, PI-based- protease inhibitor-based

^1^Variables included in the multivariable analysis: gender, weight for age, Number of regimen changes, Current ART regimen, history of tuberculosis.

#### Multivariable analysis

On multivariable analysis gender, malnutrition, and current ART regimen remained significant predictors of virologic failure. Further, the odds of failure were 60% lower for children with a history of TB compared to those who had not had TB (aOR 0.4 95% CI 0.2–0.9).

### Subsequent up viral load testing

During the follow-up period, 66 (67.3%) cases and 135 (67.2%) controls had a subsequent VL performed (p = 0.98). No child with virologic failure on first test had a repeat VL within recommended 3 month time frame and only 9 (13.6%) had a repeat VL performed in less than 6 months. Median time to subsequent VL was 14.4 months (95%CI 13.8–14.8) for suppressed children and 11.3 months (95% CI 9.5–13.2) for unsuppressed children. Among children with additional viral load results, VL suppression on follow-up was greater among those suppressed at baseline 126/135 (93.3%) in comparison to children with virologic failure on initial testing 15/66 (22.7%), (p<0.0001) (Fig 2). Among the 99 children without subsequent VL, status in HIV treatment could be determined for 97 among whom 77 (79%) remained active in care, 11 (11%) were lost to follow up and 9 (9%) transferred care to another clinic. Most children (57/66) 86% with virologic failure remained on the same ART regimen and 81% (46/57) of these children remained unsuppressed. Only 9/66 (14%) unsuppressed children were changed to a second line regimen during follow up. While only 19% of children who stayed on the same regimen achieved viral suppression during follow up, 44% of children who were changed to a second line regimen did. Among children with VL > 1000 cps/ml on initial testing (n = 66), 7 (11%) were receiving a PI-based regimen designated as first-line of which 2/7 (29%) had viral suppression at follow-up, 15 were receiving a PI-based second-line regimen, of which 9/15 (60%) had viral suppression, and 44 (67%) were receiving an NNRTI-based regimen, of which only 4/44 (9%) achieved viral suppression at follow-up. The adjusted odds of continued virologic failure was 90% lower for children on PI-based regimen for second line treatment compared to those on an NNRTI-based regimen, (aOR 0.1, 95% CI 0.0–0.4) after controlling for missed clinic visits, history of opportunistic infections and adherence support intensification but did not remain significant for those on PI-regimen as 1^st^ line.

## Discussion

Our study documents the results from routine viral load monitoring among HIV-positive children on ART in western Kenya. We found a high adoption of initial viral load testing (94%) but lower rates of subsequent testing (67%). Less than two-thirds of children on ART in this setting were suppressed at time of initial routine viral load, similar to other cohorts in resource-limited settings [5]. Male gender, malnutrition, and being on a second-line regimen were predictive of failure to achieve viral suppression. Very few children with virologic failure achieved suppression after at least 12 months of follow up, however those switched to a PI-based regimen were much more likely to achieve suppression.

While routine viral load testing is recommended for children on ART by WHO, access to this expensive and resource-intensive laboratory test remains suboptimal. According to a 2014 WHO survey of LMICs, only 20% of patients on ART receive a viral load test [25]. Between January and June 2016, Kenya reported viral load uptake of 49% among all people on ART of 49%, down from 76% in 2015 [20]. Kenya is relatively well resourced with adequate country-wide capacity for routine and targeted viral load testing. The decrease in uptake in the first half of 2016 is attributed to the upsurge in numbers of patients on ART owing to the Kenya Ministry of Health’s adoption of WHO’s 2015 recommendations to begin all persons diagnosed with HIV on ART regardless of CD4 count (i.e. test-and-start). Weak sample transportation networks, cold chain logistical challenges, staffing shortages, equipment limitations and health worker training gaps have all been reported as major barriers to viral load uptake [26]. Our study shows successful adoption of initial routine viral load among children on ART, but poorer viral load monitoring thereafter, especially for children with virologic failure. For children who have viral suppression on the initial viral load test, a follow up test is recommended at 12 months—one third of children in the present study did not receive the recommended follow-up test, including children with virologic failure on initial testing. This may have been related to an emphasis nationally to obtain routine viral load testing for all patients on ART with less guidance on subsequent testing and management of children with virologic failure. Current Kenya National ART guidelines provide additional guidance and tools on the management of virologic failure to address this gap. Children with virologic failure who did have additional viral load testing mostly remained unsuppressed. No child with virologic failure on initial viral load had a subsequent test at the recommended three months. This is likely related to the stipulation per National Guidelines that subsequent testing for those with virologic failure should be done only after adherence is clearly improved. However, there was not clear guidance on adherence interventions, nor strong adherence assessment strategies in place nationally during the study period. These findings highlight that while capacity to perform VL is available, translating this capacity to achieving virologic suppression for children remains a challenge. A recent *Medecins Sans Frontiers* report on improving implementation of viral load monitoring in LMIC emphasizes the need for systems that flag patients in need of VL testing, assigning clinic-based point people to champion VL testing and perform enhanced adherence counseling as well as demand creation and education among patients [27].

Virologic suppression rates for children on ART among LMICs range from 40–90%, demonstrating that there is much work to be done to reach the third “90” goal. [27–30] Our study findings concur with the suboptimal rates of virologic suppression in children on ART and highlight challenges with management of children with virologic failure. We found that less than one in four children with virologic failure achieved virologic suppression during follow up. Few studies document outcomes for children with elevated viral loads in routine programmatic settings. One study in Swaziland [29] found children were significantly less likely to re-suppress than adults even if they received a targeted adherence intervention. Barriers to virologic suppression in children may include poor adherence due to poor palatability of drugs and reliance on caregivers to give medications, pre-treatment drug resistance, complex weight-based dosing, and lack of health care worker confidence in treating children with HIV [31]. While current WHO and in-country guidelines suggest adherence interventions for children with treatment failure, there is no consensus on effective adherence interventions [32]. There is, therefore, an urgent need to identify evidence-based adherence interventions to prevent and address treatment failure among children in LMIC. There is also a need to build the capacity of health care workers to provide timely interventions in cases of confirmed treatment failure.

Drug resistance in children with virologic failure may compromise the success of any adherence interventions. Several studies show that 60–90% of children who fail to achieve viral suppression have at least one resistance mutation, including PI mutations [31,33–36]. Resistance testing remains costly and inaccessible but may become more important for children failing on ART in the long term. In Mali, baseline NNRTI resistance was common in children without reported PMTCT drug exposure and was associated with increased risk of treatment failure [33]. Reports from Central African Republic [35] and Kenya [34] show drug resistance in as high as 90% of children with detectable viral loads. We found few children achieved virologic suppression after failure was detected but the odds of virologic suppression were significantly higher for children on a PI-based versus NNRTI regimen. These findings warrant further exploration to support health care workers to manage children with virologic failure. In the absence of drug resistance information, close monitoring, multidisciplinary support and prompt clinical judgment are key in ensuring children who have failed treatment are appropriately transitioned to second line therapy.

Our study identified several potential risk factors for failing to achieve virologic suppression among children on ART including male gender, malnutrition, and type of drug regimen. Male children in our study were twice as likely to have a high viral load at routine viral load testing. This contrasts with a recent study from Tanzania in which female children had more than twice the risk of virologic failure than males and warrants further investigation [36]. There is mounting evidence that food insecurity is associated with incomplete viral suppression among children and adults on ART [37]. In this study, severely and moderately malnourished children were less likely to suppress. Malnutrition can impair the immune response and contribute to HIV disease progression even in children on antiretroviral therapy. Children with a history of tuberculosis had better virologic outcomes. This may be due to the close monitoring, frequent clinic visits and adherence support, including directly observed therapy that is provided as part of tuberculosis treatment. We found discrepant results regarding drug regimen type and virologic suppression. While children who were on second-line ART at time of initial viral load testing were four times more likely to have virologic failure as compared to those on a first-line regimen, on subsequent viral load testing, those on 2^nd^ line ART were much more likely to be suppressed than those on 1^st^ line regimens. This is likely related to ongoing adherence issues in these children rather than challenges with palatability or dosing of PI-based regimens as those children on PI regimen as first line were not more likely to fail. This is at odds with findings for children during follow up where children on PI-based 2^nd^ line regimen were much more likely to achieve viral suppression after failure was detected compared to those on an NNRTI-based regimen. However, due to the discrepant results, we are unable to conclude that switch to PI-based therapy alone, will lead to viral suppression. Given the high rates of transmitted and acquired NNRTI resistance among children on ART, it is possible switching to second line while also providing enhanced adherence counseling and monitoring, facilitated these children to achieve viral suppression [15,36,38,39]. (We were unable to detect associations between self-reported adherence, missed visits, or CD4 count on virologic failure highlighting the importance of routine VL testing to identify children in need of intervention.

The findings from this study show the challenges experienced in achieving virologic suppression for children in routine settings in Kenya. We were able to follow a cohort of children on ART to determine long-term outcomes for children and risk factors associated with failure to achieve viral suppression. While children in Kenya are on ART regimens recommended by WHO, their outcomes and risk factors may not be generalizable to other settings. Our cohort is from urban, high-volume clinics which may not be representative of children seen at rural or low-volume clinics. We were not able to obtain drug resistance testing, impact of prior PMTCT drug exposure, strong adherence assessments, family dynamics, socioeconomic status or other psychosocial factors that may influence viral suppression in children due to the use of routinely collected data. Additionally, while adherence intensification was standard at all sites, it was not clear defined nor routinely documented and therefore not possible to measure how often it is completed and its impact on subsequent viral suppression.

## Conclusion and recommendations

To achieve UNAIDS 90-90-90 goal for children an additional one million children will need to be identified and initiated on ART by 2020. Considerable increases in access to routine viral load monitoring will be required to ensure optimal management of these children and those already receiving treatment. Additional implementation studies are required to determine successful packages of interventions that will successfully address virologic failure in children if we are to achieve the final “90” for children living with HIV.

## Supporting information

S1 Dataset Journal Dataset_final (1).xlsx. This is the de-identified data from study participants.
(XLSX)
