# Correction: Adoption of routine virologic testing and predictors of virologic failure among HIV-infected children on antiretroviral treatment in western Kenya

**DOI:** 10.1371/journal.pone.0210908

**Published:** 2019-01-10

**Authors:** Julie Kadima, Elizabeth Patterson, Margaret Mburu, Cinthia Blat, Marilyn Nyanduko, Elizabeth Anne Bukusi, Craig Cohen, Patrick Oyaro, Lisa Abuogi

The fifth author’s first name is incorrect. The correct name is: Marilyn Nyanduko.
